# CDC25 Inhibition in Acute Myeloid Leukemia–A Study of Patient Heterogeneity and the Effects of Different Inhibitors

**DOI:** 10.3390/molecules22030446

**Published:** 2017-03-11

**Authors:** Annette K. Brenner, Håkon Reikvam, Kristin Paulsen Rye, Karen Marie Hagen, Antonio Lavecchia, Øystein Bruserud

**Affiliations:** 1 Section for Hematology, Department of Clinical Science, Faculty of Medicine and Dentistry, University of Bergen, Bergen 5021, Norway; annette.brenner@uib.no (A.K.B.); Kristin.Rye@uib.no (K.P.R.); marie.hagen@uib.no (K.M.H.); 2 Department of Medicine, Haukeland University Hospital, Bergen 5021, Norway; Hakon.Reikvam@uib.no; 3 “Drug Discovery” Laboratory, Department of Pharmacy, University of Naples Federico II, Naples 80131, Italy

**Keywords:** CDC25 inhibitors, acute myeloid leukemia, gene expression, cytogenetics, leukemic cell differentiation

## Abstract

Cell division cycle 25 (CDC25) protein phosphatases regulate cell cycle progression through the activation of cyclin-dependent kinases (CDKs), but they are also involved in chromatin modulation and transcriptional regulation. CDC25 inhibition is regarded as a possible therapeutic strategy for the treatment of human malignancies, including acute myeloid leukemia (AML). We investigated the in vitro effects of CDC25 inhibitors on primary human AML cells derived from 79 unselected patients in suspension cultures. Both the previously well-characterized CDC25 inhibitor NSC95397, as well as five other inhibitors (BN82002 and the novel small molecular compounds ALX1, ALX2, ALX3, and ALX4), only exhibited antiproliferative effects for a subset of patients when tested alone. These antiproliferative effects showed associations with differences in genetic abnormalities and/or AML cell differentiation. However, the responders to CDC25 inhibition could be identified by analysis of global gene expression profiles. The differentially expressed genes were associated with the cytoskeleton, microtubules, and cell signaling. The constitutive release of 28 soluble mediators showed a wide variation among patients and this variation was maintained in the presence of CDC25 inhibition. Finally, NSC95397 had no or only minimal effects on AML cell viability. In conclusion, CDC25 inhibition has antiproliferative effects on primary human AML cells for a subset of patients, and these patients can be identified by gene expression profiling.

## 1. Introduction

Acute myeloid leukemia (AML) is an aggressive malignancy characterized by the accumulation of immature myeloblasts in the bone marrow. The median age at diagnosis is about 65 years; in most cases, AML occurs de novo, but it can also be secondary to chemotherapy or develop from myelodysplastic syndromes (MDS) or a chronic myeloproliferative neoplasia [1]. Even though AML is highly heterogeneous with regard to its predisposition, as well as its cell morphology, cytogenetics, and gene mutations, only the acute promyelocytic leukemia (APL) variant entails its own treatment regime [2]. More than 50% of AML patients carry clonal chromosomal abnormalities [3], which can imply favorable, intermediate, or adverse prognosis. Furthermore, AML is associated with certain gene mutations, with FMS-related tyrosine kinase 3 internal tandem duplications (*Flt3*-ITD; adverse prognosis) and nucleophosmin (*NPM1*; favorable prognosis) mutations being the most significant [4]. The backbone of AML treatment for younger patients involves initial intensive induction treatment with cytarabine plus an anthracyclin, followed by intensive consolidation chemotherapy [3,5,6], but even for these patients, the relapse risk is still relatively high and the overall 5-year survival rate is less than 50%. Elderly patients cannot receive the most intensive and potentially curative regime, due to the risk of treatment-related mortality [3,7]. Thus, a need for patient-adjusted treatments has arisen. 

Cell division cycle 25 (CDC25) dual-specificity protein phosphatases are cell cycle regulators that also seem to be involved in chromatin modulation and transcriptional regulation [8]; they activate cyclin-dependent kinases (CDKs), which are important regulators of cell cycle progression [9]. Mammalian cells express three different CDC25 proteins, CDC25A, -B, and -C, all of which have been implicated in G_2_/M progression. At the G_2_/M boundary, CDC25B has been identified as the trigger phosphatase that initially activates CDK1/cyclin B, which then activates CDC25C, resulting in an auto-activation loop that drives entry into mitosis [10,11,12,13]. While CDC25A is predominantly recognized for its involvement in earlier phases of cell cycle progression, it has also been shown to play a role at the G_2_/M transition [14,15] and is the only CDC25 family member which is embryonically lethal in knockout mice [16]. In this respect, the loss of CDC25A leads to cell cycle arrest during the differentiation of malignant hematopoietic cells [17], and a reduction in CDC25B expression in AML cells also has antiproliferative effects [18]. In contrast to other phosphatases, the active site of CDC25s is flat and shallow, which makes it more challenging to design suitable inhibitors [9]. Despite this, a wide range of inhibitor chemotypes have been described and recently reviewed [9,19,20,21,22], but the majority can be categorized into three classes: phosphate bioisosteres, electrophilic entities, and quinone-based inhibitors. It is thought that there are three possible mechanisms through which these molecules inhibit CDC25s, i.e., reversible inhibition by binding to the active site of CDC25s [23,24,25,26], irreversible inhibition by covalent bond formation [27,28], or oxidation of the critical cysteine residue in the catalytic domain (CX_5_R).

**Figure 1:**
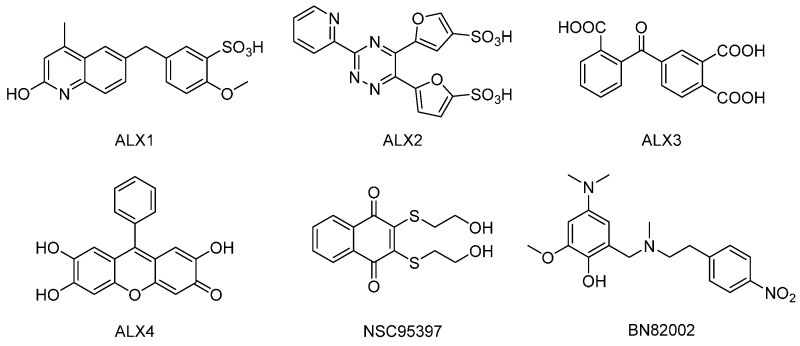
Chemical structures of cell division cycle 25 (CDC25) inhibitors used in the study: 5-((2-hydroxy-4-methylquinolin-6-yl)methyl)-2-methoxybenzenesulfonic acid (ALX1), 5,5′-(3-(pyridin-2-yl)-1,2,4-triazine-5,6-diyl)-difuran-2-sulfonic acid (ALX2), 4-(2-carboxybenzoyl)-phthalic acid (ALX3), 2,6,7-trihydroxy-9-phenyl-3*H*-xanthen-3-one (ALX4), 2,3-bis-((2-hydroxyethyl)thio)-1,4-naphthoquinone (NSC95397) and 4-dimethylamino-2-methoxy-6-((methyl-(2-(4-nitrophenyl)ethyl)amino)methyl)phenol (BN82002).

Lavecchia et al. have previously reported the discovery of new structurally distinct classes of CDC25 inhibitors with cellular activity, through structure-based high-throughput virtual screening [29], as well as by using multiple ligand-based chemoinformatic approaches [30]. The 5-((2-hydroxy-4-methylquinolin-6-yl)methyl)-2-methoxybenzenesulfonic acid (ALX1, see Figure 1), the 5,5′-(3-(pyridin-2-yl)-1,2,4-triazine-5,6-diyl)-difuran-2-sulfonic acid (ALX2), the 4-(2-carboxybenzoyl)phthalic acid (ALX3), and the 2,6,7-trihydroxy-9-phenyl-3*H*-xanthen-3-one (ALX4), had in vitro IC_50_ values in the micromolar concentration range against the purified recombinant forms of the catalytic domains of CDC25A, -B, and -C. Unlike ALX4, which inhibited the three CDC25 proteins in a non-competitive manner, ALX1, ALX2, and ALX3 caused the reversible inhibition of CDC25B and displayed competitive inhibition kinetics. These compounds affected cell cycle progression and showed significant growth inhibition against breast (MCF-7), prostate (PC-3), and leukemia (K562) cancer cell proliferation.

CDC25 is now considered to be a possible prognostic marker and/or therapeutic target, both in solid tumors and hematological malignancies [30,31,32,33,34,35,36]. First, a recent study suggested that CDC25 expression is a prognostic marker associated with chemoresistance in lung cancer, and this effect may be due to an interaction with the *c-Myc* oncogene [31]. Second, another study proposed that CDC25 causes STAT5 (signal transducer and activator of transcription 5) activation and thereby becomes a regulator of both AML cell proliferation and differentiation, at least for a subset of patients [32]. Finally, several new CDC25 inhibitors with effects on human malignant cells have recently been identified [30,33,34,35,36]. Taken together, these studies strongly suggest that CDC25 inhibition should be further investigated as a possible strategy for the treatment of human AML, and these studies have to include comparisons of different inhibitors and different patient subsets. 

In this study, the effect of ALX1, ALX2, ALX3, and ALX4 was tested on AML blasts derived from 79 consecutive AML patients (clinical and biological characteristics are given in Supplementary Table S1), and were compared with the effect of the naphthoquinone NSC95397, the most potent CDC25 inhibitor to date [25], and the phenol BN82002, which strongly inhibits CDC25 activation, delays cell cycle progression at the G_1_/S transition, in S phase, and at the G_2_/M transition, and induces apoptosis [37,38]. The cell cycle is intertwined with the phosphatidylinositol 3-kinase/Akt and mammalian target of rapamycin (PI3K/Akt/mTOR) pathways [39]; CDC25B seems able to rescue the mTOR pathway upon rapamycin treatment [40] and silencing of CDC25s can block the activation of Akt [41]. In the present study, we therefore investigated the effects of CDC25 inhibition on AML cell viability, proliferation, and constitutive cytokine release; however, CDC25 inhibition was also combined with the mTOR complex 1 (mTORC1)-targeting rapamycin and the PI3K-targeting GDC0941. Finally, we compared the global gene expression profiles for responders and non-responders to CDC25 inhibition. Our studies suggest that CDC25 inhibition is effective for a subset of AML patients, and this subset can be identified from the patients’ gene expression profile.

## 2. Results

### 2.1. CDC25 Inhibition has Antiproliferative Effects in Primary AML Cells for a Subset of Patients

Cytokine-dependent—granulocyte macrophage colony stimulating factor (GM-CSF), stem cell factor (SCF), and Flt3 ligand (Flt3L)—AML cell proliferation in suspension cultures was investigated for all 79 patients, and proliferation was assayed as (^3^H)-thymidine incorporation after seven days of in vitro culture, when the proliferative response reflects an enrichment of clonogenic cells [42]. We first analyzed the effects of the six CDC25 inhibitors for all 79 patients; these results are summarized in Table 1. A median (^3^H)-thymidine incorporation, corresponding to at least 1000 counts per minute (cpm), was regarded as detectable proliferations [43]. When comparing the overall results, only ALX4 showed a statistically significant antiproliferative effect (Wilcoxon’s signed rank test, *p* = 0.012). 

**Figure 2:**
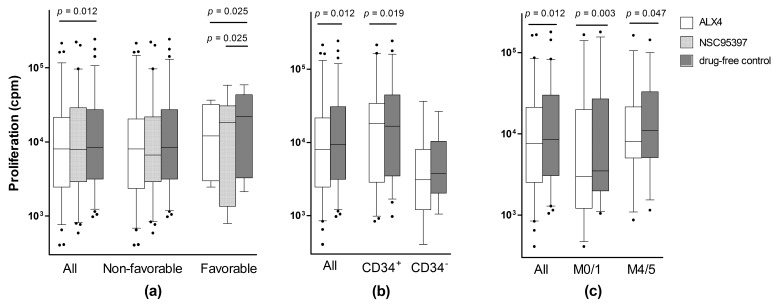
Effects of CDC25 inhibitors on cytokine-dependent (granulocyte macrophage colony-stimulating factor, stem cell factor and FMS-related tyrosine kinase 3 ligand) AML cell proliferation. Primary AML cells were cultured in suspension cultures prepared with medium alone or in medium supplemented with ALX4, at a final concentration of 10 μM. Cytokine-dependent AML cell proliferation was assayed as (^3^H)-thymidine incorporation after seven days of culture (i.e., (^3^H)-thymidine added on day six); a proliferation at this time after the initiation of culture reflects an enrichment of clonogenic cells [42]. The results are presented as the nuclear incorporation of radioactivity (counts per minute, cpm), and the figure shows box plots (median and quartiles), together with the 90% confidence intervals and outliers. All statistically significant differences are indicated at the top of the figures. (**a**) The effect of CDC25 inhibition on AML cell proliferation was analyzed for all AML cells (left), cells with non-favorable cytogenetics (middle), and cells with favorable cytogenetics (right); for each of these three comparisons, the results for ALX4 (white), NSC95397 (light grey), and drug-free control cultures (dark grey) are presented; (**b**) The effect of ALX4 on AML cell proliferation was also compared for all patients and patients with CD34^+^/CD34^−^ leukemic cells. The figure compares the results for cultures prepared with (white) and without ALX4 (grey); (**c**) The effect of ALX4 on AML cell proliferation, a comparison of all patients, patients showing no/few morphological signs of differentiation (corresponding to FAB-M0/1), and patients showing monocytic differentiation. For each of these three analyses, the figure presents the results for cultures with (white) and without ALX4 (grey).

We then investigated whether the effects of the various CDC25 inhibitors differed among AML subsets, i.e., patients having AML cells with and without morphological or molecular signs of differentiation (FAB classification, expression of the hematopoietic progenitor cell antigen CD34), or different cytogenetic or molecular genetic abnormalities (*Flt3*-abnormalities, *NPM1* insertions). Both ALX4 and NSC95397 showed statistically significant differences in the strength of the antiproliferative effects, when comparing patients with (eight patients) and without favorable cytogenetics (Figure 2a; Wilcoxon’s test, *p* = 0.025 for both drugs). Furthermore, for ALX4, the antiproliferative effect was stronger for CD34^+^ than for CD34^−^ AML cell populations (Figure 2b; *p* = 0.019), and for AML cells with no/minor morphological signs of differentiation (Figure 2c; FAB-M0/1 versus all others, *p* = 0.003). For the other CDC25 inhibitors, no significant differences were detected with respect to differentiation or genetic abnormalities (data not shown). 

We finally used the χ^2^ likelihood ratio test as an alternative statistical analysis of the pharmacological effects in the various patient subsets. The pharmacological effects were then categorized as <20%, 20%–50%, and >50% inhibition of AML cell proliferation, compared with the corresponding control cultures. The same significant differences with regard to the genetics/differentiation described above were detected by these alternative analyses (data not shown). 

To conclude, the antiproliferative effects of CDC25 inhibition differ among patients and patient subsets, identified based on genetic abnormalities and AML cell differentiation; however, it should also be emphasized that the differences between these subsets are relatively small (see Figure 2).

### 2.2. Identification of a Patient Subset Showing Growth Inhibition by Various CDC25 Inhibitors 

**Figure 3:**
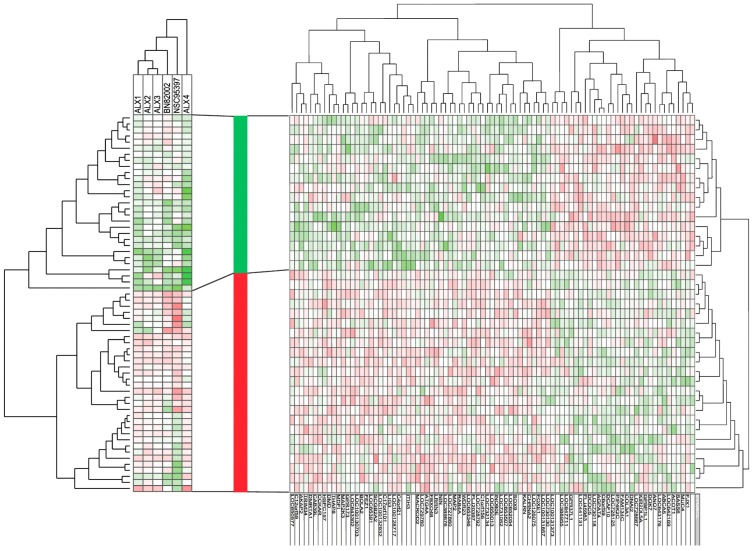
Comparison of gene expression profiles for primary human AML cells—a comparison of AML cell populations with and without an antiproliferative effect of CDC25 inhibitors. (LEFT) Cells derived from 62 AML patients were cultured in suspension cultures ((^3^H)-thymidine incorporation assay), in combination with six different CDC25 inhibitors (ALX1-4, BN82002 and NSC95397). Only patients without the t(15;17) cytogenetic aberration and with proliferation above >1000 cpm, both in control and drug-containing cultures, were included in this analysis. The (^3^H)-thymidine incorporation (cpm) for drug-containing cultures was made relative to the incorporation for corresponding drug-free controls and thereafter log_2_ converted. These values were then used for an unsupervised hierarchical cluster analysis (weighted pair group method with averaging, Euclidian distance measure). Green color represents an inhibitory and thus antiproliferative effect. (MIDDLE) Based on this analysis, the patients could be divided into two main subsets, as indicated by the bar between the two main parts of the figure; responders showing antiproliferative effects (green part of the column) and non-responders with no antiproliferative effect and even showing growth enhancement (red part of the column) in the presence of CDC25 inhibitors. (RIGHT) Global gene expression profiles were available for 39 unselected patients (16 with antiproliferative and 23 without antiproliferative effects of CDC25 inhibition). Patients with and without antiproliferative effects were compared by a feature subset analysis, and 87 differentially expressed genes (*p* < 0.003) were detected. These 87 genes could be used for a hierarchical cluster analysis (weighted pair group method with averaging, Euclidian distance measure) that divided the patients into two main subsets, corresponding to the 16 patients with and the 23 patients without an antiproliferative effect induced by CDC25 inhibitors. Green indicates upregulated genes and red indicates downregulated genes.

Instead of analyzing the antiproliferative effects of each individual drug, we completed an unsupervised hierarchical clustering, based on the effects of all six CDC25 inhibitors on the cytokine-dependent in vitro proliferation of primary human AML cells derived from patients without the t(15;17) cytogenetic aberration. In this analysis, we only included patients showing AML cell proliferation corresponding to at least 1000 cpm, both in the control cultures and in all of the drug-containing cultures (for all detectable values see Table 1); this was done to ensure that we could estimate the percent alteration of the proliferative response for all patients and drugs, without making adjustments based on our definition of detectable proliferation as corresponding to >1000 cpm. The results are presented in Figure 3 (left part, 62 patients included). The effect of the CDC25 inhibitors differed among patients and drugs. Firstly, two major patient subsets could be identified, based on the antiproliferative effect of the CDC25 inhibitors, and antiproliferative effects were especially seen for the lower subset. Secondly, ALX1, ALX2, and ALX3 clustered together and showed similarities with regard to the antiproliferative effects, whereas the other three drugs (BN82002, NSC95397, and ALX4) clustered separately (Figure 3, see top of the left part). These two main patient subsets did not differ significantly with regard to genetic abnormalities (cytogenetic abnormalities; *NPM1* and *Flt3* mutations) or AML cell differentiation (FAB classification, CD34 expression). 

### 2.3. Responders to CDC25 Inhibition Can Be Identified by Analysis of Gene Expression Profiles

Global gene expression profiles were available for an unselected subset of 39 patients. These patients included 16 patients showing a relatively strong growth inhibition by CDC25 inhibitors (included in the lower main cluster in the left part of Figure 3), and 23 patients showing weak/no antiproliferative effects (included in the lower main cluster in Figure 3, left part). We identified 87 genes (of which six are not annotated) that differed significantly between the two patient subsets, identified in the pharmacological clustering (Figure 3, left part). The functions of the encoded proteins are summarized in Table 2 and a detailed description is given in Supplementary Table S2. A major part of the genes encodes proteins that are important for the function of the cytoskeleton and/or the microtubule system, i.e., they are involved in the regulation of cell cycle progression/mitosis. Thereafter, we did an unsupervised hierarchical clustering of these patients, based on the expression of the 87 identified genes; the clustering analysis based on this limited number of genes identified two main patient subsets (Figure 3, right part) that corresponded to the patient subsets included in the lower/upper patient clusters, respectively, from the pharmacological clustering in Figure 3, left part. Thus, an analysis of gene expression profiles can be used to identify patients that differ in their susceptibility to CDC25 inhibitors. 

### 2.4. Combined CDC25 and PI3K/mTOR Inhibition Has Additive Antiproliferative Effects Only for Subsets of Patients 

Previous studies have demonstrated that CDC25B expression is associated with resistance to the antiproliferative effect of PI3K/Akt/mTOR inhibitors [40] and that CDC25B activity is required for the activation of Akt, whereas the inhibition/knockdown of CDC25A reduces Akt-dependent AML cell proliferation [44]. Therefore, we investigated the antiproliferative effects of PI3K/Akt/mTOR inhibitors. Both the PI3K inhibitor GDC0941 and the mTORC1 inhibitor rapamycin caused a highly significant inhibition of AML cell proliferation when analyzing the overall results (Table 1, *p* < 0.001). The effect of combining GDC0941 and rapamycin with 10 μM of the CDC25 inhibitors NSC95397 (79 consecutive patients) and ALX4 (23 consecutive patients), was also tested. The strong antiproliferative effects induced by the PI3K inhibitor GDC0941 and the mTORC1 inhibitor rapamycin, were maintained in the presence of the CDC25 inhibitors, but no general additive or synergistic effects were detected. 

We also used the χ^2^ likelihood tests to investigate the additive or synergistic antiproliferative effects in patient subsets, and NSC95397 plus rapamycin (i.e., an mTORC1 inhibitor) had stronger antiproliferative effects than rapamycin alone for CD34^+^ cells/patients (*p* = 0.006). A stronger inhibition was also seen for patients with favorable cytogenetics (*p* = 0.003), both when comparing (i) patients with/without the inv(16) cytogenetic aberration, and (ii) the four subsets with favorable/normal/intermediate/adverse cytogenetics. This is similar to the results for CDC25 inhibition (see above and Figure 2), where the antiproliferative effects were stronger for AML cells expressing CD34 or those which had a favorable karyotype. In contrast, the effect of NSC95397 plus GDC0941 (a PI3K inhibitor) did not show similar associations with CD34 expression or karyotype (data not shown). This difference between the effects of the two combination therapies may be caused by the different effects of the two drugs on Akt activation, due to the inhibition of pathway mediators upstream (i.e., PI3K) or downstream (mTORC1) to Akt, respectively [6].

### 2.5. CDC25 Inhibition Has Only Minor Effects on the Constitutive Cytokine Release by AML Cells

Primary AML cells derived from the 79 patients were cultured in suspension cultures prepared in medium alone and medium supplemented with 10 μM NSC95397; supernatants were harvested after 48 h and the levels of 28 soluble mediators then determined. The constitutive release in control cultures showed a wide variation among patients, for all of the mediators (values are provided in Supplementary Table S3). It can also be seen that there was a wide variation among patients with respect to the mediator levels in NSC95397-containing cultures, and that all mediators except the chemokine CXCL1 and the angiopoietin receptorTie-2, showed statistically significant correlations between levels in control cultures and in the drug-containing cultures. Thus, the differences in cytokine release profiles between individual patients are maintained in the presence of NSC95397. 

### 2.6. CDC25 Inhibition Does Not Alter the Viability of In Vitro Cultured AML Cells 

AML cell viability was determined by flow-cytometry using the Annexin V-propidium iodide apoptosis assay, and as expected [45], there was a wide variation in viability after 40 h of in vitro culture in medium alone (median viability 36%, range 2%–81%). The presence of 10 μM NSC95397 during culture had no significant effect on viability, neither when analyzing the overall results for the 79 patients (median 35%, range 6%–80%), nor when comparing various patient subsets that differed with regard to AML cell differentiation (morphology according to the FAB classification, expression of the CD34 stem cell marker), cytogenetic abnormalities, or *Flt3/NPM1* mutations (data not shown). Finally, AML cell viability was also tested after culture with 10 μM ALX4; we then examined primary cells derived from 23 consecutive patients. Again, there was a wide variation in viability in medium alone (median 38%, range 1%–81%), and no significant difference upon the presence of ALX4 (median 40%, range 1%–87%) (data not shown).

## 3. Discussion

In this study, we investigated the effect of six CDC25 inhibitors on primary human AML cells; these cells were derived from a large group of unselected patients. Our in vitro studies showed that (i) these drugs only had antiproliferative effects for a subset of patients; (ii) the growth inhibition showed no strong associations with parameters commonly used for the subclassification of AML patients (genetic abnormalities, differentiation); (iii) but instead, the responders could be identified based on their gene expression profiles. Taken together, these observations suggest that future clinical studies of CDC25 inhibition in human AML should possibly focus on the identification of patient subsets with increased susceptibility to this therapeutic strategy. Finally, even though CDC25 inhibitors affected AML cell proliferation, we could not detect any general effect of these agents on the stress-induced or spontaneous in vitro apoptosis. The explanation for this discrepancy is probably that the percentage of apoptotic/necrotic cells is determined by the viability of the more mature majority of leukemic cells that only survive for a limited time during in vitro culture [45]. In contrast, proliferation after seven days of culture (i.e., (^3^H)-thymidine added at day six) is determined by a more immature progenitor cell subset which is able to survive and proliferate for a longer time in vitro, and the ability to continue to proliferate after seven days of in vitro culture thereby reflects an enrichment of the clonogenic AML cell subset [42]. Furthermore, in a previous study of ALX1-3 and their anti-malignant effect on cell lines, much higher concentrations than the IC_50_ dose were required in order to significantly reduce cell viability, e.g., for ALX3 (IC_50_ approximately 10 μM), 200 μM had to be added [29]. 

In our present study, we investigated patients with high peripheral blood AML blast counts and/or a high percentage of circulating leukemic cells among the blood leukocytes. This selection of patients was used because highly enriched leukemic cell populations could then be prepared with gradient separation alone, for all of the included patients. Previous studies have described that functional alterations can be induced in AML cells when more extensive separation procedures are used [42,46]. Even though a previous study showed that the frequencies of the important prognostic parameters (i.e., genetic abnormalities associated with chemoresistance/chemosensitivity) did not differ between the overall AML patient populations and the patients selected according to these criteria [47], our observations should be interpreted with care and may be representative only for this selected subset of patients. However, the independent prognostic impact of high peripheral blood blast count seems to be relatively weak and is seen mainly for certain patient subsets. High peripheral blood blast counts are often defined as >100 × 10^9^/L [48,49,50]. The number of patients with such high blast counts was relatively small among our patients, and when taking into account that we included a relatively large number of elderly patients with an expected low frequency of favorable karyotype, the frequency of patients with high blood blast counts in our present study is comparable with previous studies [50]. Taken together, these observations suggest that our patients are representative for the overall leukemia cell population with regard to chemosensitivity/chemoresistance.

We used cryopreserved primary AML cells in our study. Even though these cells show a decreased viability compared with fresh leukemic cells, their viability was generally above 70% upon thawing, and it was thereby possible to analyze alterations in viability during short-term in vitro culture. By using cryopreserved cells, it was possible to investigate cells from different patients in each experiment and the inter-assay variation could thereby be reduced. However, one should emphasize that the viability decreases during the first two to four days of culture [45], so our viability studies probably reflect the characteristics of another AML cell subset (i.e., the majority of short-living cells in a two days assay), rather than our proliferation assay (i.e., a minority of enriched clonogenic cells were able to survive and still proliferate after seven days of culture). 

CD34 expression was analyzed by flow cytometry. We then used >20% stained cells as the definition of CD34 positivity. This cut-off is generally accepted and commonly used [51], although certain studies suggest the use of a higher cut-off value [52]. 

The growth inhibitory effect of CDC25 targeting showed an association with favorable cytogenetic abnormalities; these abnormalities are associated with a strong antileukemic effect of cytarabine-based treatment [53,54]. Our observations may therefore suggest that the mechanisms behind the resistance to CDC25 inhibition are at least partly overlapping with the mechanisms behind cytarabine resistance. 

We also combined CDC25 inhibition with PI3K/Akt/mTOR targeting. These overall results suggest that the antiproliferative effects of both CDC25 and PI3K/Akt/mTOR inhibition are strongest for AML cells having favorable cytogenetic abnormalities and/or showing less signs of differentiation, judged from the high expression of the CD34 stem cell marker and/or having few morphological signs of differentiation (i.e., FAB classification FAB-0/1/2; absent monocytic differentiation). A similar observation was described by Didier et al. [55]: an increased antileukemic effect by the checkpoint 1 inhibitor UCN-01 (7-hydroxystaurosporine) and the topoisomerase II inhibitor etoposide, was observed for AML cells without morphological signs of differentiation (defined as FAB-M1/2) versus cells with monocytic differentiation (FAB-M4/5). Thus, all of these different pharmacological strategies (inhibition of CDC25, mTORC1, Checkpoint 1 or Topoisomerase II) seem to have stronger effects for less differentiated cells. In contrast, the combination of CDC25 inhibition, plus the PI3K inhibitor GDC0941, did not show the same associations with karyotype/differentiation. This discrepancy in antiproliferative pharmacological effects between CD34^+^ and CD34^−^ may be caused by differences in the expression of CDC25 [39,55]; but a more likely explanation seems to be that the pharmacological effects depend on the biological/intracellular context of CDC25 [55]. 

As described by Didier et al. [55], the susceptibility to certain antileukemic treatment seems to depend on the differentiation status of the AML cells, and their studies also suggest that drug-induced growth inhibition may be mediated by other mechanisms than drug-induced apoptosis. CD25A and CD25B both seem to be increased in human AML cells [39]. CDC25B can be upregulated in AML cells upon DNA damage, which seems to cause growth recovery in CD34^+^ cells, whereas CD34^−^ AML cells entered apoptosis [55]. Such heterogeneity within the AML cell populations may explain why some patients are not susceptible to CDC25 inhibition. It may also explain why the drug effects on proliferation (i.e., long-term surviving/proliferating cells) and viability (short-term assay) differ.

The differences between the ALX compounds regarding their mode of action and more remote cellular effects, might also help to explain why ALX4 stood out, and why it had an anti-proliferative effect on a subgroup of patients. First, ALX1-3 cause reversible, competitive CDC25 inhibition with ALX1 and ALX2 mainly targeting CDC25A, whereas ALX3 also inhibits CDC25B [29]. It seems likely, on the other hand, that ALX4, like a series of its analogues, is an irreversible, non-competitive inhibitor of CDC25s [29,30], thus making it a more potent inhibitor. Second, the drugs differ in their binding to CDC25. Whereas ALX1-3 binds to the so-called “swimming-pool”—an area adjacent to the active site—in addition to the catalytic pocket, ALX4 and its derivatives appear to only bind to the catalytic site [29]. The latter is assumed to allow for all three possible CDC25 inhibition mechanisms, i.e., covalent modification of the enzyme, oxidation, and inactivation of the catalytic cysteine, either through redox cycling and/or the production of reactive oxygen species [29]. In that respect, an increased production of reactive oxygen species was observed for both ALX4 and its analogues [29,30]. Third, the ALX4-derivative 2-(6-hydroxy-3-oxo-3*H*-xanthen-9-yl)cyclohexane- 1-carboxylic acid also reduced the amount of phosphorylated Akt compared to total Akt, and increased the amount of p53 [30]. The deactivation of Akt and its downstream targets might at least partly explain why the addition of mTORC1-inhibitor rapamycin only increased the anti-proliferative effect of the CDC25 inhibitor NSC95397 for the patient subgroup which already showed an anti-proliferative response upon drug-induced CDC25 inhibition.

A recent study described that CDC25A is only an important regulator of AML cell proliferation and differentiation for leukemic cells with *Flt3*-ITD, but not for cells with the wildtype gene [32]. The authors further described that. in a cohort of 100 samples from AML patients with intermediate-risk cytogenetics, high CDC25A mRNA levels only predicted a higher clonogenic potential in *Flt3*-ITD samples, and the CDC25 inhibitor IRC-083864 only had an antiproliferative effect and only induced monocytic differentiation for *Flt3*-ITD, but not for *Flt3*-wt AML cells. However, we could not observe any association between the antiproliferative effect of CDC25 inhibition and *Flt3*-ITD. Several explanations are possible for this discrepancy between the previous study and our present observations. First, when we examined a panel of CDC25 inhibitors, we observed different effects of various inhibitors, even when testing the same patient. Second, our highly standardized proliferation assay is based on suspension cultures in serum-free medium, without the addition of conditioned medium. Third, we investigated a larger group of consecutive, and thereby more heterogeneous, patients. However, both studies suggest that the therapeutic targeting of CDC25 will only be effective in selected AML patients. One possible explanation for the differences between patients could be variances in the levels of the CDC25A/B/C isoforms. Finally, both studies also suggest that there is a link between monocytic differentiation and an antiproliferative effect upon CDC25 inhibition. 

We analyzed the global gene expression profiles for an unselected subset of patients, and by comparing responders and non-responders to CDC25 inhibition, we identified a subset of differentially expressed genes. The function is unknown for a relatively large number of these genes, but a majority encodes proteins which are important for (i) cell cycle progression, i.e., direct cell cycle regulation, DNA repair, cytoskeleton/microtubule function, and intracellular trafficking; or (ii) intracellular signaling, especially the ubiquitination of proteins, G protein coupled receptor signaling, or regulation of transcription (Table 2). Our observations thus suggest that gene expression/mRNA profiling (i.e., a method suitable for routine clinical practice) can be used to identify responders to CDC25 inhibition. 

Previous functional in vitro studies in other cell types have demonstrated that the effects of CDC25 inhibition are associated with the acetylation/modulation of tubulin [56,57]; our present results also suggest that responsiveness to the CDC25 inhibitors depends on the expression of genes that encode regulators of the cytoskeleton/microtubules. This association between the effects of CDC25 inhibitors and the expression of these regulators is probably due to their role as downstream or indirect targets during CDC25 inhibition. In our opinion, it seems less likely that chemically/pharmacologically different CDC25 inhibitors should have direct common off-target effects on these regulatory molecules. 

We compared the effect of the CDC25 inhibitor NSC95397 on the constitutive release of 28 soluble mediators by primary human AML cells. These cytokines were selected because they are commonly released by primary human AML cells [40,58,59]. The cytokine release showed a very wide variation among patients (Supplementary Table S3); this was especially true for several chemokines. This wide variation with significant correlations between the levels in drug-free and drug-containing cultures was maintained for most mediators in the presence of CDC25 inhibition, and NSC95397 only caused a relatively small alteration for a small minority of the 28 mediators. Taken together, our observations show that the individual differences among patients with respect to the constitutive cytokine release persist in the presence of CDC25 inhibition and are probably more important than the relatively weak effects of NSC95397 on the release of a minority of soluble mediators. 

In conclusion, our in vitro studies of six CDC25 inhibitors suggest that CDC25 inhibition only has antiproliferative effects for a subset of AML patients, and that ALX4 seems to be particularly effective. Our results also suggest that the analysis of gene expression (mRNA) profiles in the primary AML cells may be a useful tool for the identification of responders to this therapeutic strategy. 

## 4. Materials and Methods 

### 4.1. AML Patient Population and Cell Isolation

The study was approved by the local Ethics Committee (Regional Ethics Committee III, University of Bergen, Bergen, Norway; REK VEST 2013/634, April 15^th^ 2013) and samples were collected after written informed consent. AML blasts were derived from the peripheral blood of 79 consecutive patients (Supplementary Table S1) with either high peripheral blood blast counts or a high percentage of AML cells in the peripheral blood. For certain experiments, a group of 23 consecutive patients was tested. Due to the selection of patients with high peripheral blood blast counts, enriched AML cells could be isolated by density gradient separation alone (Lymphoprep, Axis-Shield, Oslo, Norway; specific density 1.077 g/mL), and these cell populations contained at least 90% AML blasts based on flow-cytometric estimations. Twenty-seven patients had total white blood cell counts exceeding 100 × 10^9^/L, but for ten of these patients, a borderline value of total leukocytes below 105 × 10^9^/L was seen at the time of sampling, and the peripheral blood AML blast count was then below 100 × 10^9^/L. The large majority of contaminating cells were small lymphocytes. 

Cells were stored in liquid nitrogen until used [60]. All AML cells were frozen and thawed, according to the same highly standardized methods [46,61]. Generally, they had a viability exceeding 70%, based on staining with trypan-blue immediately after thawing, and the viability then gradually decreased over the next two to four days, due to spontaneous in vitro apoptosis [45]. 

### 4.2. Reagents 

The culture medium was Stem Span SFEM medium (Stem Cell Technologies, Vancouver, BC, Canada). NSC95397 and BN82002 (Calbiochem; Merck Millipore, Darmstadt, Germany), as well as the trial compounds ALX1, ALX2, ALX3, and ALX4, were examined at a final concentration of 10 μM. This concentration has been used in previous studies for NSC95397 and BN82002 [38,58]. For the trial compounds, we additionally performed dose-response examinations (100, 50, 25, 10, 5, 2.5, and 1 μM) of their effects on cytokine-dependent AML cell proliferation (four patients examined together with the AML cell lines KG-1a, CTV-1, EOL, and HL60), and at 10 μM, the antiproliferative effects of these drugs (as well as NSC95397 and BN82002) had reached a plateau for those patient cells/AML cell lines that were susceptible to CDC25 inhibition. This concentration was therefore used in our experiments to detect differences among cells/patients in the susceptibility to growth inhibition induced by CDC25 inhibition. GDC0941 (Axon Medchem, Groningen, The Netherlands) and rapamycin (Sigma Aldrich, St. Louis, MO, USA) were used at a final concentration of 0.1 μM; this concentration can also be used to detect differences among patients [40]. 

### 4.3. Analysis of AML Cell Apoptosis and CD34 Expression by the Leukemic Cells

AML cells (2 × 10^5^ cells/well) were cultured in flat-bottomed microtiter plates (200 μL/well; Costar, Cambridge, MA, USA). All cell cultures were incubated at 37 °C in a humidified atmosphere of 5% CO_2_. After 40 h, the percentage of viable cells was determined by flow cytometry, subsequent to staining with propidium iodide and fluorescein isothiocyanate-conjugated Annexin V (Tau Technologies BV, Kattendijke, The Netherlands) [62]. 

Blast CD34 expression was analyzed by flow cytometry, and the cells were regarded as CD34 positive when at least 20% of the cells were stained, compared with an isotype control [51]. 

### 4.4. Analysis of Spontaneous and Cytokine-Dependent Proliferation

AML cells (5 × 10^4^ cells/well) were cultured in flat-bottomed microtiter plates (200 μL/well) in Stem Span for six days, before 37 kBq/well of (^3^H)-thymidine (Perkin Elmer, Waltham, MA, USA) was added to each well and the nuclear radioactivity was determined 24 h later. Thus, this assay reflects an ability of cells to continue proliferation after more than six days of culture, and this proliferation therefore reflects an enrichment of clonogenic cells [42]. Cells were either cultured in medium alone (spontaneous/autocrine proliferation) or in medium supplemented with 20 ng/mL of GM-CSF, SCF, and Flt3L. 

### 4.5. Analysis of Cytokine Levels in Culture Supernatants

Supernatants were collected from AML cells cultured for 48 h in Stem Span (1 × 10^6^ cells per mL) in 24-well culture plates (Nunclon, Roskilde, Denmark). The samples were stored at −80 °C, until analyzed. Cytokine levels were determined by Luminex analyses (R & D Systems, Minnesota, MN, USA) or by enzyme-linked immunosorbent assays (ELISA, R & D Systems; Cusabio, Wuhan, China). The following cytokines were investigated: (i) the chemokines CXCL1/2/5/8/10/11 and CCL2-5; (ii) the interleukins IL-1β/1RA/6/10/33; (iii) the matrix metalloproteinases MMP-1/2/9 and their antagonist, the tissue inhibitor of metalloproteinases 1 (TIMP-1); (iv) the immunomodulatory tumor necrosis factor-α (TNFα); (v) the growth factors granulocyte colony stimulating factor (G-CSF), GM-CSF, hepatocyte growth factor (HGF), heparin-binding EGF-like growth factor (HB-EGF), basic fibroblast growth factor (bFGF), vascular endothelial growth factor (VEGF) and angiopoietin 1 (Ang-1), and (vi) the angiopoietin receptor Tie-2 (tyrosine kinase with immunoglobulin-like and EGF-like domains 2).

### 4.6. RNA Preparation and Microarray Analysis

Global gene expression profiles were analyzed using the Illumina iScan Reader (Illumina Inc., San Diego, CA, USA), based on the fluorescent detection of biotin-labeled complementary RNA (cRNA). Briefly, 300 ng total RNA from each sample was reversely transcribed, amplified, and labeled with Biotin-16-UTP using the Illumina TotalPrep RNA Amplification Kit (Applied Biosystems/Ambion, Foster City, CA, USA). The amount and quality of the biotin-labeled cRNA was controlled by both a NanoDrop spectrophotometer and an Agilent 2100 Bioanalyzer (Agilent Technologies, Santa Clara, CA, USA), before 750 ng of biotin-labeled cRNA was hybridized to the HumanHT-12 V4 Expression BeadChip (Illumina Inc.), according to the manufacturer’s instructions. The chip targets 47,231 probes derived primarily from genes in the NCBI RefSeq database (Release 38) [40]. 

### 4.7. Bioinformatic and Statistical Analyses

Bioinformatical analyses were performed using the J-Express 2012 software (MolMine AS, Bergen, Norway). The data from array scanning were quantile normalized and log_2_ transformed. A feature subset selection (FSS) analysis was performed using *t*-score and individual ranking as parameters. Scores with an absolute fold change of at least 3.0 were regarded as statistically significant. The statistical analyses were performed with the IBM Statistical Package for the Social Sciences (SPSS) version 23 (Chicago, IL, USA). The Wilcoxon signed rank test was used to evaluate drug effects, whereas χ^2^ tests (likelihood ratio due to few patients in the subgroups) were used for correlation analyses. *p*-values < 0.05 were regarded as statistically significant. 

## Figures and Tables

**Table 1:** The effects of CDC25 inhibition of acute myeloid leukemia (AML) cell proliferation in suspension cultures. Proliferation was measured as (^3^H)-thymidine incorporation after seven days of in vitro culture. The table presents the number of patients with detectable proliferation (>1000 cpm), both in drug-free and corresponding drug-containing cultures, the median proliferation in drug-containing cultures for these patients and the corresponding variation range for these patients with detectable proliferation, and finally, the *p*-values when comparing proliferation in cultures with CDC25 inhibitors with the corresponding CDC25 inhibitor-free control cultures.

	Proliferative AML Cell Responses Corresponding to Activity >1000 cpm			
Drugs Added	Number ^a^	Median (cpm)	Range (cpm) ^a^	*p*-Value ^b^
Drug-free control	69	6929	1149–244,316	–
NSC95397	64	6349	1045–223,371	Ns
BN82002	69	5586	1094–260,005	Ns
ALX1	67	5888	1051–220,146	Ns
ALX2	68	6937	1151–213,939	Ns
ALX3	68	6393	1062–180,671	Ns
ALX4	64	6005	1051–215,655	0.012
GDC0941	69	4580	1053–202,884	<0.001
NSC95397 + GDC0941	67	3892	1049–170,748	<0.001
Rapamycin	61	3665	1030–105,449	<0.001
NSC95397 + Rapamycin	60	3626	1032–142,996	<0.001

^a^ Number of patients with detectable proliferation (defined as >1000 cpm) in the control or the corresponding drug-containing cultures; ^b^ Ns, not statistically significant (Wilcoxon’s signed rank test, *p* > 0.05) when comparing the overall results.

**Table 2:** Analysis of global gene expression profiles—a comparison of responders and non-responders to CDC25 inhibitors. A total of 81 annotated genes showed differential expression, 41 of which had a known function.

Functional Classification	Number	DOWN-Regulated mRNA Expression in Responders	UP-Regulated mRNA Expression in Responder
**Cell cycle, mitosis** **DNA repair**	4	SASS6 (centrosome),	NIN (centrosome) FEZ1 (centrosome) BOLA2 (cell cycle regulation)
**Cytoskeleton, microtubule**	8	DNAI2 (centrosome, spindle formation) DCDC5 (tubulin binding)	FLJ20397 (trafficking, organelle positioning, microtubule organization) WDR23 (actin cytoskeletal organization; **ubiquitination**) MAP2 (microtubule assembly) DMD (cytoskeletal) AKAP2 (anchoring protein) LIG3 (DNA repair)
**Intracellular trafficking**	2		KALRN (vesicle trafficking) RAB4A (**GTPase**, endosomes)
Cell membrane molecules Extracellular molecules	8	MUC4 (glycoprotein) ABCA5 (transmembrane transport intra- and extracellular) KIR2DL5A (cell surface molecule) COL3A1 (collagen)	GPR173 (**G protein coupled receptor**) CCKAR (**G protein coupled receptor**) ITIH3 (matrix stabilization) CHRNA2 (ion channel)
Intracellular signaling	10	FAM134C PIP4K2C (PI3K signalling, endoplasmic reticulum) DCAF10 (**ubiquitin**) GPR37L1 (**G protein coupled receptor**) DOK7 (kinase phosphorylation)	CIorf156 (methyltransferase) WDR23 (**ubiquitination**) FBXO48 (**ubiquitination**) MAP2K3 (MAP kinase) CAB39L (LKB1 activation) TMEM52B (**ubiquitination**)
Transcription	6	GPBP1L1	FOXE1, SOX9, INO8OE, MDFI, DMRTA1 (transcriptional regulators)
Metabolism, autophagy	4	ACOT1 (metabolism, acetyl-CoA) AGPAT4 (metabolism, phospholipid biosynthesis)	ATG4A (autophagy) MACROD2 (apoptosis)
Unknown	40	FJX1, LOC643176, ANO7, LOC728667, LOC728125, C6orf59, LOC730118, FLJ45983, LOC441131, LOC647711, LOC388955, LOC100131373	LOC730130, LOC131857, LOC126075, LOC652054, LOC653507, LOC731052, LOC650013, LOC732134, LOC728792, LOC653346, LOC727860, LOC389676, LNNR3, LOC729260, ZGRF1, LOC100128717, OGFOD3, LOC642169, LOC100132932, SCGB2A2, LOC85391, LOC100130703, LOC643302, THAP8, HSPC157, TRIM34, LOC650577

## Supplementary Materials

The following are available online. Table S1: Clinical and biological characteristics of the 79 AML patients included in the study, Table S2: Single genes identified in the bioinformatical comparison of responders versus non-responders to CDC25 inhibitors, Table S3: Constitutive cytokine release by primary human AML cells—the effect of 10 μM NSC95397.
