# Supplementary material for: CDC25 Inhibition in Acute Myeloid Leukemia–A Study of Patient Heterogeneity and the Effects of Different Inhibitors
Source: Molecules. 2017 Mar 11;22(3):446. doi: 10.3390/molecules22030446 (PMC6155411; doi:10.3390/molecules22030446)
Supplement: Supplementary file 1 [file molecules-22-00446-s001.pdf]

Supplementary Materials: CDC25 Inhibition in Acute Myeloid Leukemia−A Study of Patient Heterogeneity and the Effects of Different Inhibitors

Annette K. Brenner, Håkon Reikvam, Kristin Paulsen Rye, Karen Marie Hagen, Antonio Lavecchia and Øystein Bruserud

Table S1. Clinical and biological characteristics of the 79 AML patients included in the study.

**Patient characteristics**		**Cell morphology**		**Cell genetics**	
Age		*FAB classification*		*Cytogenetic*[3]	
Median (years)	67	M0	6	Favorable	9
Range (years)	18-77	M1	21	Intermediate	6
		M2	11	Normal	41
Gender		M3	2	Adverse	15
Females	34	M4	18	n.d.	8
Males	45	M5	15		
		n.d.[2]	6	*Flt3 mutations*	
Secondary AML[1]				ITD[4]	28
MDS	8	*CD34 expression*		Wild-type	37
Chemotherapy	1	Negative (≤ 20 %)	21	n.d.	14
CM(M)L	4	Positive (> 20 %)	53		
		n.d.	5	*NPM1 mutations*	
AML relapse[1]	6			Insertion	26
				Wild-type	40
				n.d.	13

[1] Thirteen patients had secondary AML and six patients relapsed AML (two of them relapse of secondary AML).
[2] n.d.: not determined
[3] 15 patients had an adverse cytogenetic abnormalities (i.e. −5; −7; +8; complex karyotype) whereas the nine patients in the favorable risk group had inv(16) (six patients), t(15;17) (two patients) and t(8;21) (one patient).
[4] One of the patients has an additional point mutation at D835.

Table S2. Single genes identified in the bioinformatical comparison of responders versus non-responders to CDC25 inhibitors. A total of 81 annotated genes were identified, and only 46 of them have a known function. A major subset of 18 genes encode proteins that are directly involved in cell cycle regulation (1 gene), the function of microtubuli/cytoskeleton (9 genes) and intracellular organelle formation/trafficking (4); 4 additional genes are involved in protein ubiquitination that is important for regulation of cell cycle progression/CDC25 modulation. These genes may thus represent a functional entity that is important for cell cycle progression/mitosis. The remaining genes are transcriptional regulators together with a DNA ligase (6 molecules), surface membrane molecules or surface membrane receptors (7 molecules), and molecules involved in intercellular communication (2 genes) or intracellular signaling (3 molecules). Thus, none of the molecules involved in regulation of the early steps of the cell cycle showed differential expression, but several genes important for mitosis/cell division differed between the two groups.

**DOWNREGULATED IN RESPONDERS TO CDC25 INHIBITORS**		
FJX1	Four jointed box 1	Important for embryonic development in Drosophila, unknown function in humans.
MUC4	MUCIN 4	Encoding an integral membrane glycoprotein found on the cell surface.
SASS6	SAS-6 centriolar assembly protein	SAS6 is necessary for centrosome duplication and functions during procentriole formation; SAS6 functions to ensure that each centriole seeds the formation of a single procentriole per cell cycle.
ACOT1	Acyl-CoA thioesterase 1	Cell metabolism.
LOC643176		
ABCA5	ATP-binding cassette, sub-family A, member 5	A membrane-associated protein that is a member of the superfamily of ATP-binding cassette transporters (ABC1 subfamily). These proteins transport various molecules across extra- and intracellular membranes.
LOC642169		
DOK7	Docking protein 7	The encoded protein can induce autophosphorylation of receptor kinase; the protein may also be implicated in breast cancer development [1, 2].
ANO7	ANOCTAMIN 7	The protein is a member of the anoctamin family; these proteins are proposed to have eight transmembrane domains with both termini facing the cytoplasm and a C-terminal domain of unknown function.
GPBP1L1	GC-rich promoter binding protein 1-like 1	
KIR2DL5A	Killer cell immunoglobulin-like receptor, two domains, long cytoplasmic tail, 5	Killer cell immunoglobulin-like receptors (KIRs) are transmembrane glycoproteins classified by the number of extracellular immunoglobulin domains (2D or 3D) and by whether they have a long (L) or short (S) cytoplasmic domain. KIR proteins with the long cytoplasmic domain transduce inhibitory signals upon ligand binding.
LOC728667		
DNAI2	Dynein, axonemal, intermediate chain 2	Belongs to the dynein intermediate chain family and part of the dynein complex of respiratory cilia and sperm flagella (see also FLJ20397). Cytoplasmic dynein is a large minus-end-directed microtubule motor complex, involved in many different cellular processes including intracellular trafficking, organelle positioning, and microtubule organization. Furthermore, dynein plays essential roles during cell division where it is implicated in multiple processes including centrosome separation, chromosome movements, spindle organization, spindle positioning, and mitotic checkpoint silencing [3].
COL3A1	Collagen, type III, alpha 1	This gene encodes the extracellular fibrillar pro-alpha1 chains of type III collagen.
FAM134C	Family with sequence similarity 134, member C	A nuclear respiratory factor-1 (NRF-1) transcriptional target expressed in neuroblastoma cells, regulates cellular outgrowth.

PIP4K2C	Phosphatidylinositol-5-phosphate 4-kinase, type II, gamma	The protein has similarity to phosphatidylinositol 5-phosphate 4-kinase IIalpha and IIbeta isoforms, may play a role in phosphatidylinositol bisphosphate synthesis in the endoplasmic reticulum.
LOC728125		
DCAF10 (WDR32)	DDB1 and CUL4 associated factor 10	Possibly involved in ubiquitination [4].
C6orf59	AGPAT4 intronic transcript	Unknown
AGPAT4	1-acylglycerol-3-phosphate O-acyltransferase 4	This gene encodes a member of the 1-acylglycerol-3-phosphate O-acyltransferase family. This integral membrane protein converts lysophosphatidic acid to phosphatidic acid, the second step in de novo phospholipid biosynthesis.
LOC730118		
DCDC5	Doublecortin domain containing 5	This gene encodes a doublecortin family protein, i.e. a tubulin-binding protein that enhances microtubule polymerization.
FLJ45983	GATA3 antisense RNA 1	The gene can show altered methylation in breast cancer.
LOC441131		
GPR37L1	G protein-coupled receptor 37 like 1	An orphan G protein-coupled receptor, its ligand prosaposin (also known as sulphated glycoprotein-1) promotes its endocytosis, induces phosphorylation of ERK, stimulates (35)S-GTPγS binding, inhibits cAMP production and protects against oxidative stress [5].
LOC647711		
LOC388955		
LOC100131373		
**UPREGULATED IN RESPONDERS TO CDC25 INHIBITION**		
LOC730130		
LOC131857		
FOXE1	Forkhead box E1provided	A member of the forkhead family of transcription factors.
LOC126075		
CHRNA2	cholinergic receptor, nicotinic, alpha 2	Nicotinic acetylcholine receptors (nAChRs) are ligand-gated ion channels, neurotransmitter receptor.
KALRN	kalirin, RhoGEF kinase	May have a function in vesicle trafficking.
SOX9	SRY (sex determining region Y)-box 9	A DNA binding and transcription-regulating protein
LOC652054		
LOC653507		
LOC731052		
INO80E	INO80 complex subunit	Possibly involved in chromatin remodelling/transcriptional regulation [6].
LOC650013		
LOC732134		
C1orf156	Methyltransferase like 18	Possibly important for platelet function/thrombus formation? [7].
LOC 728792		

FLJ20397	Dynein, axonemal, assembly factor 5	The protein is essential for the preassembly or stability of axonemal dynein arms (see also DNAI2 above). Cytoplasmic dynein is a large minus-end-directed microtubule motor complex, involved in many different cellular processes including intracellular trafficking, organelle positioning, and microtubule organization. Furthermore, dynein plays essential roles during cell division where it is implicated in multiple processes including centrosome separation, chromosome movements, spindle organization, spindle positioning, and mitotic checkpoint silencing [3].
LOC653346		
WDR23	DDB1 and CUL4 associated factor 11	This gene encodes a WD repeat-containing protein that interacts with the COP9 signalosome, a macromolecular complex that interacts with cullin-RING E3 ligases and regulates their activity by hydrolysing cullin-Nedd8 conjugates. Cullin-Ring E3 ubiquitin ligases are important for actin cytoskeletal organization [8].
RAB4A	RAB4A, member RAS oncogene family	Belongs to the Ras superfamily of small GTPases, it is associated with early endosomes and is involved in their sorting/recycling and recycling of receptors from endosomes to the plasma membrane.
MAP2	Microtubule-associated protein 2	Belongs to the microtubule/cytoskeletal-associated protein family, possibly involved in microtubule assembly.
LOC727860		
LOC389676		
NIN	Ninein (GSK3B interacting protein)	This gene encodes a protein important for centrosomal function and for positioning and anchoring of microtubules
LNNR3	Liver neoplastic nodule remodelling QTL 3	Unknown
FBXO48	F-box protein 48	This is a member of the F-box protein family (Fbxs subclass) that is characterized by an approximately 40 amino acid motif referred to as the F-box; these proteins constitute one of the four subunits of the ubiquitin protein ligase complex called SCFs (SKP1-cullin-F-box) involved in phosphorylation-dependent ubiquitination.
ATG4A	Autophagy related 4A, cysteine peptidase	This gene encodes a member of the autophagin protein family that is important for autophagy, the protein also being designated as a member of the C-54 family of cysteine proteases.
LOC729260		
MACROD2	MACRO domain containing 2	The protein can mediate chemoresistance in breast cancer cells [9].
ITIH3	Inter-alpha-trypsin inhibitor heavy chain 3	This gene encodes the heavy chain subunit of the pre-alpha-trypsin inhibitor complex; this complex may stabilize the extracellular matrix through its ability to bind hyaluronic acid.
ZGRF1 (C4orf21)	Zinc finger, GRF-type containing 1	Unknown
LOC100128717		
LIG3	Ligase III, DNA, ATP-dependent	This gene is a member of the DNA ligase family, encoding a protein that catalyses the joining of DNA ends, being involved in excision repair and being located in both mitochondria and nucleus
OGFOD3 (C17orf101)	2-oxoglutarate and iron-dependent oxygenase domain containing 3	Unknown
LOC100132932		
SCGB2A2 (MGB1)	Secretoglobin, family 2A, member 2	The protein can be expressed in breast cancer cells
LOC85391		

FEZ1	Fasciculation and elongation protein zeta 1	CLASP2 and NEK1 proteins are present in a centrosomal complex and participate in cell cycle and cell division mechanisms, and they co-localize with FEZ1 in a perinuclear region and interact with endogenous gamma-tubulin [10].
BOLA2	BolA family member 2	The protein belongs to the family of BolA-like proteins probably involved in cell proliferation or cell-cycle regulation.
LOC100130703		
LOC643302		
GPR173	G protein-coupled receptor 173	This gene encodes a member of the G-protein coupled receptor 1 family.
MAP2K3	Mitogen-activated protein kinase 3	The protein belongs to the MAP kinase family. It phosphorylates and thus activates MAPK14/p38-MAPK.
MDFI	MyoD family inhibitor	This protein is a transcription factor.
THAP8	THAP domain containing 8	Unknown
DMD	Dystrophin	Dystrophin is a large cytoskeletal protein, it is part of the dystrophin-glycoprotein complex (DGC) that bridges the inner cytoskeleton (F-actin) and the extra-cellular matrix, it seems to interact with microtubules and have a role in cell division/cell cycle regulation, and it is expressed in hematopoietic cells [11, 12].
HSPC157	Long intergenic non-protein coding RNA 33	Unknown
CCKAR	Cholecystokinin A receptor	This gene encodes a G-protein coupled receptor.
CAB39L	Calcium binding protein 39-like	CABL39L can activate the LKB1 tumour suppressor [13].
DMRTA1	Doublesex and mab-3 related transcription factor like family A1	The protein is a Pax6 activated transcription factor [14].
TRIM34	tripartite motif containing 34	The protein is a member of the tripartite motif (TRIM) family, the TRIM motif including three zinc-binding domains, a RING, B-box type 1 and B-box type 2 domain, and a coiled-coil region. Unknown functions.
AKAP2	A kinase (PRKA) anchor protein 2	The encoded protein binds to the regulatory subunit of protein kinase A and is found associated with the actin cytoskeleton.
TMEM52B (C12orf59)	Transmembrane protein 52B	The protein is possibly involved in protein ubiquitination [15].
LOC650577		

References to Table S2

1. Fitzgerald, L. M.; Browne, E. P.; Christie, K. D.; Punska, E. C.; Simmons, L. O.; Williams, K. E.; Pentecost, B. T.; R, M. J.; Otis, C. N.; Arcaro, K. F., ELF5 and DOK7 regulation in anti-estrogen treated cells and tumors. *Cancer Cell Int* **2016,** *16*, 8.
2. Heyn, H.; Carmona, F. J.; Gomez, A.; Ferreira, H. J.; Bell, J. T.; Sayols, S.; Ward, K.; Stefansson, O. A.; Moran, S.; Sandoval, J.; Eyfjord, J. E.; Spector, T. D.; Esteller, M., DNA methylation profiling in breast cancer discordant identical twins identifies DOK7 as novel epigenetic biomarker. *Carcinogenesis* **2013,** *34*, (1), 102-108.
3. Raaijmakers, J. A.; Medema, R. H., Function and regulation of dynein in mitotic chromosome segregation. *Chromosoma* **2014,** *123*, (5), 407-422.
4. Bennett, E. J.; Rush, J.; Gygi, S. P.; Harper, J. W., Dynamics of cullin-RING ubiquitin ligase network revealed by systematic quantitative proteomics. *Cell* **2010,** *143*, (6), 951-965.
5. Meyer, R. C.; Giddens, M. M.; Schaefer, S. A.; Hall, R. A., GPR37 and GPR37L1 are receptors for the neuroprotective and glioprotective factors prosaptide and prosaposin. *Proc Natl Acad Sci U S A* **2013,** *110*, (23), 9529-9534.
6. Chen, L.; Cai, Y.; Jin, J.; Florens, L.; Swanson, S. K.; Washburn, M. P.; Conaway, J. W.; Conaway, R. C., Subunit organization of the human INO80 chromatin remodeling complex: an evolutionarily conserved core complex catalyzes ATP-dependent nucleosome remodeling. *J Biol Chem* **2011,** *286*, (13), 11283-11289.
7. Zhang, Y.; Tong, Y.; Zhang, Y.; Ding, H.; Zhang, H.; Geng, Y.; Zhang, R.; Ke, Y.; Han, J.; Yan, Z.; Zhou, L.; Wu, T.; Hu, F. B.; Wang, D.; Cheng, J., Two novel susceptibility SNPs for ischemic stroke using exome sequencing in Chinese Han population. *Mol Neurobiol* **2014,** *49*, (2), 852-862.
8. Hudson, A. M.; Mannix, K. M.; Cooley, L., Actin Cytoskeletal Organization in Drosophila Germline Ring Canals Depends on Kelch Function in a Cullin-RING E3 Ligase. *Genetics* **2015,** *201*, (3), 1117-1131.
9. Mohseni, M.; Cidado, J.; Croessmann, S.; Cravero, K.; Cimino-Mathews, A.; Wong, H. Y.; Scharpf, R.; Zabransky, D. J.; Abukhdeir, A. M.; Garay, J. P.; Wang, G. M.; Beaver, J. A.; Cochran, R. L.; Blair, B. G.; Rosen, D. M.; Erlanger, B.; Argani, P.; Hurley, P. J.; Lauring, J.; Park, B. H., MACROD2 overexpression mediates estrogen independent growth and tamoxifen resistance in breast cancers. *Proc Natl Acad Sci U S A* **2014,** *111*, (49), 17606-17611.
10. Lanza, D. C.; Meirelles, G. V.; Alborghetti, M. R.; Abrile, C. H.; Lenz, G.; Kobarg, J., FEZ1 interacts with CLASP2 and NEK1 through coiled-coil regions and their cellular colocalization suggests centrosomal functions and regulation by PKC. *Mol Cell Biochem* **2010,** *338*, (1-2), 35-45.
11. Teniente-De Alba, C.; Martinez-Vieyra, I.; Vivanco-Calixto, R.; Galvan, I. J.; Cisneros, B.; Cerecedo, D., Distribution of dystrophin- and utrophin-associated protein complexes (DAPC/UAPC) in human hematopoietic stem/progenitor cells. *Eur J Haematol* **2011,** *87*, (4), 312-322.
12. Villarreal-Silva, M.; Centeno-Cruz, F.; Suarez-Sanchez, R.; Garrido, E.; Cisneros, B., Knockdown of dystrophin Dp71 impairs PC12 cells cycle: localization in the spindle and cytokinesis structures implies a role for Dp71 in cell division. *PLoS One* **2011,** *6*, (8), e23504.
13. Fan, D.; Ma, C.; Zhang, H., The molecular mechanisms that underlie the tumor suppressor function of LKB1. *Acta Biochim Biophys Sin (Shanghai)* **2009,** *41*, (2), 97-107.
14. Sun, J.; Rockowitz, S.; Xie, Q.; Ashery-Padan, R.; Zheng, D.; Cvekl, A., Identification of in vivo DNA-binding mechanisms of Pax6 and reconstruction of Pax6-dependent gene regulatory networks during forebrain and lens development. *Nucleic Acids Res* **2015,** *43*, (14), 6827-6846.
15. Abdelmohsen, K.; Srikantan, S.; Yang, X.; Lal, A.; Kim, H. H.; Kuwano, Y.; Galban, S.; Becker, K. G.; Kamara, D.; de Cabo, R.; Gorospe, M., Ubiquitin-mediated proteolysis of HuR by heat shock. *Embo j* **2009,** *28*, (9), 1271-1282.

Table S3. Constitutive cytokine release by primary human AML cells – the effect of 10 µM NSC95397. The release of 28 soluble mediators was tested for primary human AML cells derived from 79 patients. The second column of the table shows the levels for AML cells cultured in medium alone, whereas the third column shows the levels when NSC95397 was added. The table is divided into three subgroups: cytokines that are released at high/detectable levels for a large majority of patients both in drug-containing cultures and drug-free controls (upper part); mediators that are released for most patients when leukemic cells are cultured in medium alone but at lower/undetectable levels in the presence of NSC95397 (middle); and mediators that are released only by a subset of patients also in drug-free control cultures (lower part). The right column shows the results from correlation analyses for mediator levels in drug-free control cultures and cultures with NSC95397.

	**Cytokine level medium only**			**10 µM NSC95397**			**Statistical analyses**	
**Mediator**	**No.[1]**	**Median level (pg/mL)**	**Range (pg/mL)**	**No.[1]**	**Median level (pg/mL)**	**Range (pg/mL)**	**r-value**	***p*-value**
MMP-2	79	1,288	148 – 9,485	79	1,548	172 – 12,808	0.652	< 0.001
CCL5	79	39	7.4 – 2,481	79	59	3.9 – 3,106	0.659	< 0.001
HGF	79	42	5.9 – 884	79	29	0.1– 721	0.756	< 0.001
IL-1RA	79	841	6.4 – > 10,700	77	1,296	n.d.[2] – > 10,700	0.855	< 0.001
CXCL5	78	95	n.d.[2] – > 14,500	75	150	n.d. – > 14,500	0.785	< 0.001
CXCL8	79	535	0.3 – > 18,500	77	1,312	n.d. – > 18,500	0.783	< 0.001
CXCL10	79	12	1.4 – 24,642	73	4.3	n.d. – > 25,000	0.672	< 0.001
CXCL11	79	83	25 – 246	70	26	n.d. – 95	0.178	0.036
VEGF	79	11	2.8 – 96	70	7.1	n.d. – 71	0.542	< 0.001
IL-1β	79	2.6	1.2 – 1,008	70	2.8	n.d. – 18,555	0.777	< 0.001
CCL3	79	200	119 – > 30,000	69	156	n.d. – > 30,000	0.641	< 0.001
CCL4	79	92	21 – 11,744	68	61	n.d. – > 90,000	0.753	< 0.001
MMP-1	79	33	26 – > 7,100	62	5.4	n.d. – > 7,100	0.713	< 0.001
CXCL1	79	79	41 – 22,772	58	44	n.d. – > 65,000	0.087	Ns[3]
TNFα	79	3.0	0.8 – 1,346	52	1.8	n.d. – 5,462	0.715	< 0.001
TIMP-1	79	1,812	572 – 34,789	49	852	n.d. – 102,004	0.756	< 0.001
HB-EGF	78	11	n.d. – 71	38	n.d.	n.d. – 64	0.408	< 0.001
Tie-2	79	183	21 – 248	27	n.d.	n.d. – 140	0.016	Ns
GM-CSF	79	12	8.7 – 555	26	n.d.	n.d. – 4,484	0.545	< 0.001
IL-33	79	8.5	6.6 – 22	25	n.d.	n.d. – 7.6	0.418	< 0.001
IL-10	79	1.6	0.6 – 79	19	n.d.	n.d. – 196	0.578	< 0.001
bFGF	79	19	10 – 44	17	n.d.	n.d. – 43	0.275	0.007
CXCL2	69	16	n.d. – 11,594	72	15	n.d. – 11,430	0.351	< 0.001
CCL2	67	110	n.d. – 9,807	61	170	n.d. – 6,964	0.745	< 0.001
Ang-1	61	58	n.d. – 2,135	67	98	n.d. – 1,545	0.758	< 0.001
MMP-9	47	1,545	n.d. – 261,935	45	1,192	n.d. – 316,386	0.777	< 0.001
IL-6	37	n.d.	n.d. – 19,311	60	3.7	n.d. – > 19,500	0.738	< 0.001
G-CSF	11	n.d.	n.d. – 10,262	15	n.d.	n.d. – > 10,500	0.749	< 0.001

[1] Number of patients with detectable release.

[2] n.d. below detection limit;

[3] Ns not significant.
